# Lateral Root Initiation in the Parental Root Meristem of Cucurbits: Old Players in a New Position

**DOI:** 10.3389/fpls.2019.00365

**Published:** 2019-04-10

**Authors:** Alexey S. Kiryushkin, Elena L. Ilina, Vera A. Puchkova, Elizaveta D. Guseva, Katharina Pawlowski, Kirill N. Demchenko

**Affiliations:** ^1^Laboratory of Cellular and Molecular Mechanisms of Plant Development, Komarov Botanical Institute, Russian Academy of Sciences, Saint Petersburg, Russia; ^2^Department of Ecology, Environment and Plant Sciences, Stockholm University, Stockholm, Sweden; ^3^Laboratory of Molecular and Cellular Biology, All-Russia Research Institute for Agricultural Microbiology, Saint Petersburg, Russia

**Keywords:** cucumber, squash, GATA23, lateral root initiation, MAKR4, pericycle, root meristem, xylem

## Abstract

While in most higher plants, including the model system *Arabidopsis thaliana*, the formation of lateral root primordia is induced in the elongation zone of the parental root, in seven plant families, including Cucurbitaceae, an alternative root branching mechanism is established such that lateral roots are initiated directly in the apical meristem of the parental root. In Arabidopsis, the transcription factor GATA23 and MEMBRANE-ASSOCIATED KINASE REGULATOR4 (MAKR4) are involved in the gene regulatory network of lateral root initiation. Among all marker genes examined, these are the earliest known marker genes up-regulated by auxin during lateral root initiation. In this study, putative functional orthologs of Arabidopsis *GATA23* and *MAKR4* were identified in cucumber (*Cucumis sativus*) and squash (*Cucurbita pepo*). Both cucurbits contained 26 genes encoding GATA family transcription factors and only one *MAKR4* gene. Phylogenetic and transcriptional analysis of up-regulation by auxin led to the identification of *GATA23* putative functional orthologs in Cucurbitaceae – *CpGATA24* and *CsGATA24*. In squash, *CpMAKR4* was up-regulated by naphthylacetic acid (NAA) and, similar to *MAKR4* in Arabidopsis, indole-3-butyric acid (IBA). A detailed analysis of the expression pattern of *CpGATA24* and *CpMAKR4* in squash roots from founder cell specification until emergence of lateral root primordia was carried out using promoter-fluorescent reporter gene fusions and confocal microscopy. Their expression was induced in the protoxylem, and then expanded to founder cells in the pericycle. Thus, while the overall expression pattern of these genes was significantly different from that in Arabidopsis, in founder cells their expression was induced in the same order as in Arabidopsis. Altogether, these findings suggest that in Cucurbitaceae the putative functional orthologs of *GATA23* and *MAKR4* might play a role in founder cell specification and primordium positioning during lateral root initiation. The role of the protoxylem in auxin transport as a trigger of founder cells specification and lateral root initiation is discussed.

## Introduction

During a plant’s lifetime, the development of the root system is associated with the initiation and development of lateral root primordia. In seed plants lateral roots are initiated in the pericycle, but in ferns, in the endodermis [reviewed by Charlton (1991)]. However, the radial location of the initiation site, as well as the position along the longitudinal axis of the parental root, can vary considerably (Mallory et al., 1970; Charlton, 1991; Demchenko and Demchenko, 2001; Hou et al., 2004; Ilina et al., 2018). The ancestral form of root branching is dichotomous, as shown by fossils of the ancestors of current ferns and Lycopodiophyta (Hetherington and Dolan, 2017, 2018, 2019; Liu and Xu, 2018), while in extant angiosperms, root branching is monopodial [reviewed by Motte and Beeckman (2018)]. Here, lateral roots emerge from a main axis formed by the parental roots. Thus, one of the basic questions in root evolution is how dichotomous and monopodial branching evolved.

Over the past several decades, most developmental biological studies have focused on model plant species, in the case of dicotyledonous herbs on *Arabidopsis thaliana* (hereafter Arabidopsis). Yet, model species do not encompass all types of morphogenetic mechanisms. In most higher plants, the formation of lateral root primordia is induced in the elongation zone of the parental root by a well-studied mechanism [reviewed by Du and Scheres (2018)]. However, in seven families of Angiosperms, including Cucurbitaceae, an alternative root branching mechanism is established in that lateral roots are initiated directly in the apical meristem of the parental root (Ilina et al., 2018). The study of alternative mechanisms can help us understand the evolution of root branching.

The gene regulatory network involved in lateral root initiation is well known for Arabidopsis. Key genes encoding the transcription factor GATA23, MEMBRANE-ASSOCIATED KINASE REGULATOR4 (MAKR4), and RAPID ALKALINIZATION FACTOR-like34 (RALFL34) are the earliest known markers, the transcription of which is induced during lateral root initiation in Arabidopsis (De Rybel et al., 2010; Xuan et al., 2015; Murphy et al., 2016). During founder cell specification, auxin acts through auxin regulatory components and their target genes *GATA23* and *MAKR4* (De Rybel et al., 2010; Xuan et al., 2015).

GATA transcription factors are a group of regulators that contain the highly conserved type IV zinc finger motif in the form CX_2_CX_17_–_20_CX_2_. These factors were named by their ability to bind the consensus DNA sequence (A/T)GATA(A/G). They were originally identified and characterized in animals and fungi, and are typically encoded by multi-gene families (Reyes et al., 2004). The transcription factor GATA23, which belongs to the B-class of GATA-family proteins and is specific for Brassicaceae (Behringer et al., 2014; Behringer and Schwechheimer, 2015), was found using meta-analysis of transcriptomic databases related to lateral root initiation events (De Rybel et al., 2010). In Arabidopsis, *GATA23* is specifically expressed in the primed pericycle cells prior to the first asymmetric divisions (De Rybel et al., 2010).

Priming of founder cells is a rhythmically repetitive event. Founder cell specification depends on ARF6–ARF8-mediated signaling with *GATA23* as a target (Lavenus et al., 2013). Expression levels of *GATA23* are upregulated by naphthylacetic acid (NAA) in an IAA28- and SLR/IAA14-dependent manner, indicating that auxin is directly involved in the regulation of *GATA23* expression. At the cellular level, the transcription factor GATA23 acts cell-autonomously: its activity commences in the two sister xylem pole pericycle cells before the first asymmetric division (De Rybel et al., 2010). The stages of lateral root development were defined by Malamy and Benfey (1997). Using a *pGATA23::NLS-GFP* and a *pGATA23::GATA23-GFP* fusion construct, *GATA23* expression could be detected in the pericycle up to stage II (De Rybel et al., 2010). Arabidopsis RNAi lines with 70% reduction in the expression levels of *GATA23* displayed strongly reduced numbers of lateral root primordia in stages I and II, while overexpression of *GATA23* led to an increase in the number of non-emerged primordia (De Rybel et al., 2010).

A search for proteins showing amino acid sequence similarity with the C-terminal end of the BRASSINOSTEROID KINASE INSENSITIVE 1 (BKI1) protein resulted in the identification of a new family of Arabidopsis proteins, which was named MEMBRANE-ASSOCIATED KINASE REGULATOR (MAKR) (Jaillais et al., 2011). Studies on auxin metabolism showed that one member of this family is involved in root branching. Auxin derived from the root cap due to the conversion of indole-3-butyric acid (IBA) to indole-3-acetic acid (IAA) plays an important role in the regulation of root branching (De Rybel et al., 2012). Comparisons of the transcriptome of an Arabidopsis *ibr1ibr3ibr10* triple mutant, which lacks the enzymes of the IBA-to-IAA conversion pathway, with that of the wild type after IBA treatment led to the identification of a novel IBA-regulated component of root patterning, MEMBRANE-ASSOCIATED KINASE REGULATOR 4 (MAKR4) (Xuan et al., 2015).

*MAKR4* promoter activity was found to be initially induced in the protoxylem cells within the root apical meristem, and transcription was upregulated after IAA- or IBA treatment (Xuan et al., 2015). In the Arabidopsis *ibr1ibr3ibr10* triple mutant, *MAKR4* expression levels were strongly reduced compared to the wild type even after treatment with IBA. At the cellular level, MAKR4 located to the plasma membrane and nuclei (Xuan et al., 2015; Simon et al., 2016). Roots carrying estradiol-inducible artificial microRNA constructs targeting *MAKR4* had significantly lower numbers of lateral root primordia and emerged lateral roots than wild type roots, while numbers of prebranch sites resembled those of wild type roots. Therefore, MAKR4 has been suggested to be involved in converting prebranch sites in the pericycle into a regular spacing of lateral roots (Xuan et al., 2015).

Little is known about the other members of the MAKR protein family. MAKR1, like BKI1, may interact with the BRASSINOSTEROID INSENSITIVE 1 (BRI1) receptor (Jaillais et al., 2011; Jiang et al., 2015). Unlike BKI1, a negative regulator of brassinosteroid signaling, MAKR5 is a positive effector of CLAVATA3/EMBRYO SURROUNDING REGION 45 (CLE45) signaling through the BARELY ANY MERISTEM 3 (BAM3) receptor (Kang and Hardtke, 2016). Arabidopsis *MAKR6* is one of 72 targets of the KANADI1 transcription factor, which regulates the adaxial-abaxial polarity of leaves (Xie et al., 2015). Expression of the *MAKR6* gene was upregulated in roots after 6 h of treatment with 1 μM exogenous IAA (Omelyanchuk et al., 2017).

Previously, we have shown in detail the sequence of events involved in the initiation and development of lateral root primordia in squash (*Cucurbita pepo*, Cucurbitaceae) (Ilina et al., 2018). In squash, the first symmetric anticlinal division was preceded by the formation of cellular auxin response maxima in two adjacent cells in files of the pericycle in the parental root apical meristem at a distance of 250–350 μm from the initial cells, among proliferating cells of the parental root meristem. Cellular auxin response maxima appeared at the xylem pole in pairs of sister cells (founder cells) of the three inner pericycle files, two files of the outer pericycle, and endodermis files. These observations are similar to those for Arabidopsis, where the simultaneous activation of pairs of cells took place in three files of pericycle cells at the xylem pole (Casimiro et al., 2003). Thus, the first divisions initiating lateral root formation, regardless of the place of the initiation, were anticlinal divisions in a pair of sister cells. Further development of squash lateral root primordia was associated with the involvement of three to four layers of the inner cortex. Cortex cells formed an auxin response maximum and contributed to primordium development after periclinal divisions in the pericycle and endodermis.

While in squash, exogenous application of auxin transport inhibitors led to a reduction of the number of lateral roots, exogenous auxins neither led to an increase in the total number of lateral roots nor did they affect the Dubrovsky LRI index (Ilina et al., 2018). Nevertheless, DR5 promoter mediated visualization of auxin response maxima at the earliest stages of primordium formation demonstrated a key role for endogenous auxin in lateral root initiation in squash (Ilina et al., 2018).

As already noted by Xuan et al. (2015) for Arabidopsis, so far the exact role of auxin during lateral root pre-patterning and founder cell specification remains elusive. Similarly, the genetic mechanisms leading to lateral root initiation, including founder cell specification, in Cucurbitaceae are poorly understood. In this study, we focused on two genes expressed during the initiation of lateral root primordia. In Arabidopsis, *GATA23* and *MAKR4* play a key role in specifying pericycle cells to become founder cells prior to the first formative divisions during lateral root initiation. We report the identification and expression patterns of the putative functional orthologs of *GATA23* and *MAKR4* in Cucurbitaceae.

## Materials and Methods

### Plant Material and Bacterial Strains

Squash (*Cucurbita pepo* L. var. *giromontina*) cv. Beloplodniy and cucumber (*Cucumis sativus* L.) cv. Kustovoy (Sortsemovosch, Saint Petersburg, Russia) were used in this study. *Agrobacterium rhizogenes* strain R1000-mediated transformation of squash seedlings was performed as described previously (Ilina et al., 2012). *Escherichia coli* strain XL-1 Blue was used for molecular cloning.

### Molecular Cloning

A set of genetic constructs harboring promoter–reporter fusions was developed via multisite Gateway technology using Gateway LR Clonase II Plus (Thermo Fisher Scientific, Waltham, MA, United States). To create the 242_pKGW-RR-MGW-pCpGATA24::mNeonGreen-H2B construct (*pCpGATA24::mNeonGreen-H2B*), containing the human histone *H2B* ORF (Nam and Benezra, 2009) and the 242_pKGW-RR-MGW-pCpMAKR4::eGFP-H2B construct (*pCpMAKR4::eGFP-H2B*), a series of entry vectors was generated. A list of plasmids and vectors used for entry vector construction is given in Supplementary Table S1. Constructions of binary vectors are given in Supplementary Table S2.

A set of promoter-containing entry vectors was developed containing 3036 bp of the promoter region of the *C. pepo GATA24* gene identified in this study (from –3062 to –27 before the predicted translational start site) and 2683 bp of the promoter region of the *C. pepo MAKR4* gene identified in this study (from –2688 to –6 before the predicted translational start site). Promoters were PCR-amplified using squash genomic DNA as a template, and cloned in the 369_pENTRattL4attR1_BSAI vector (Thermo Fisher Scientific) using *Sma*I restriction sites. Sequences of mNeonGreen-H2B-C6 (Shaner et al., 2013) and eGFP-H2B fusions were PCR amplified from commercial plasmid templates (Supplementary Table S1) and cloned in the pUC18-entry8 vector (Hornung et al., 2005) using *Kpn*I and *Not*I restriction sites. The *A. thaliana Actin2* gene terminator (TermAct) (Engler et al., 2014) was PCR amplified from a commercial plasmid and cloned in the 373_pENTRattR2attL3 vector (Thermo Fisher Scientific) via a Gateway BP Clonase reaction (Thermo Fisher Scientific). The resulting 373_pENTRattR2attL3-TermAct vector was used as a donor of TermAct in all further constructions.

All fusions in all constructs were verified by PCR amplification of fragments and sequencing of the products. All primers sequences are given in Supplementary Table S3. All binary vectors were introduced into *A. rhizogenes* R1000 cells by electroporation (Ilina et al., 2012).

### Plant Transformation

*Agrobacterium rhizogenes*-mediated plant transformation was carried out as described previously (Ilina et al., 2012) with several modifications. Surface sterilized seeds of squash were germinated in sterile vermiculite moistened with d H_2_O in Magenta^TM^ GA-7 vessels (Merck, Kenilworth, NJ, United States). Plants were grown in an MLR-352H incubator (Panasonic, Osaka, Japan) under controlled conditions: 16/8 h light/darkness, light intensity 600 μM/(m^2^⋅sec), humidity 96%, at 25/22°C (day/night) for seed germination and growth of transformants and at 21°C for co-cultivation of plants and agrobacteria. The first putative transformed roots were harvested approximately 2 weeks after the inoculation of squash seedlings. Further on, transformed roots 5–10 cm in length were harvested three to five times with 5-day intervals from a single transformation.

### Treatments With Exogenous Auxins

Indole-3-acetic acid (IAA, 0.3, 1, or 5 μM), naphthylacetic acid (NAA, 10 μM), and indole-3-butyric acid (IBA, 5 μM) were used as exogenous auxins. Four-day-old wild type seedlings with 5–7 cm long roots were incubated in aerated 1/4 strength Hoagland’s medium supplemented with the respective phytohormones for 6 h at 25°C during the light period. The auxins concentrations and period of exposition used in this study were selected based on data reported previously (Paponov et al., 2008; De Rybel et al., 2010; Xuan et al., 2015). The seedlings were located on a floating opaque raft, and vessels were protected by aluminum foil from light. After the treatment, root tips (1 cm from the root cap) were flash-frozen in liquid nitrogen. Frozen plant material was stored at −80°C. Each experiment included at least 25–30 seedlings and was repeated at least five times independently.

### Quantitative Real-Time PCR Assays

Total RNA was extracted from frozen plant material using the RNeasy Plant Mini Kit (QIAGEN, Hilden, Germany). To assess the integrity of the total RNA, an aliquot of each RNA sample was run on an 1% agarose gel following by staining with ethidium bromide. The quantity of each RNA sample was measured using a Qubit 2.0 fluorometer (Thermo Fisher Scientific) using the Qubit RNA BR Assay Kit.

Total RNA (1 μg) was used for reverse transcription with the Maxima First Strand cDNA synthesis kit for RT-qPCR with dsDNase (Thermo Fisher Scientific). Reverse transcription conditions were as follows: dsDNase treatment at 37°C for 10 min; addition of reverse transcription components to the same tube; incubation for 10 min at 25°C followed by 15 min at 50°C; the reaction was terminated by incubation at 85°C for 5 min. 0.4 μl of cDNA from a non-diluted sample (total volume 20 μl) was used for each qPCR reaction.

The RT-qPCR analysis was performed using an Eco Real-Time PCR system (Illumina, San Diego, CA, United States). Each qPCR reaction was carried out in a total volume of 10 μl. For *GATA* genes, detection based on SYBR Green I dye chemistry was used (Maxima SYBR Green/ROX qPCR master mix (2X), Thermo Fisher Scientific). PCR conditions were as follows: 1 cycle of 95°C for 10 min; 40 cycles of 95°C for 15 s, 52°C (for cucumber) or 60°C (for squash) for 30 s, and 72°C for 30 s; followed by reheating of PCR products at 95°C and then by starting a gradual temperature decrease from 95 to 55°C with a step of −0.3°C per s. For the squash *MAKR4* gene, detection based on TaqMan chemistry was used (Maxima Probe/ROX qPCR master mix (2X), Thermo Fisher Scientific). PCR conditions were as follows: 1 cycle of 95°C for 10 min; 40 cycles of 95°C for 15 s, 60°C for 30 s, and 72°C for 30 s. A TaqMan probe carrying carboxyfluorescein (FAM) as a fluorophore and Black Hole Quencher1 (BHQ1) as a quencher was used. All primers and probes used for qPCR are listed in Supplementary Table S4. Primers and probes were designed using the Vector NTI Advance v11.0 software (Thermo Fisher Scientific). Purified PCR primers were purchased from Evrogen (Moscow, Russia). TaqMan probes were synthetized by BioBeagle (Saint Petersburg, Russia). Each experiment was carried out with at least five biological replicates and three technical replicates. The specificity of the amplified qPCR products was verified by sequencing.

Quantification cycles (Cq) were determined using the Eco Real-Time PCR System software v4.1.11.2 (Illumina). Relative transcript levels were calculated using the 2^−ΔΔCT^ method (Livak and Schmittgen, 2001). PCR efficiency for all primer pairs was between 93 and 98%. Elongation factor *EF1a* was chosen as reference gene according to literature data about the stability of reference gene expression in cucumber (Wan et al., 2010) and squash (Obrero et al., 2011).

Plots for qPCR data were prepared using the R software default code for the boxplot function (R Core Team, 2017). Statistical analysis of the data was performed with Wilcoxon’s test from the base R package. Differences with *P*-values <0.05 were considered statistically significant. RT-qPCR analysis of the relative expression levels of *GATA* genes was performed with five biological replicates. For *MAKR4*, this analysis was performed using four to eight biological replicates.

### Phylogeny and Bioinformatics

Arabidopsis GATA and MAKR proteins from TAIR ^1^ (Berardini et al., 2015) were used as query to find amino acid sequences of *C. sativus* (cucumber, Chinese long v. 2 and Gy14) (Li et al., 2011; Yang et al., 2012), *Cucumis melo* (melon) (Garcia-Mas et al., 2012), *Citrullus lanatus* (watermelon, 97103) (Guo et al., 2013), *Cucurbita moshata*, and *Cucurbita maxima* (pumpkin) (Sun et al., 2017), *Lagenaria siceraria* (bottle gourd) (Wu et al., 2017), and *C. pepo* (squash) (Montero-Pau et al., 2018) in the Cucurbit Genomics Database ^2^. For the cucumber GATA and MAKR proteins, searches in other databases were also used: Phytozome ^3^ (Goodstein et al., 2012), NCBI ^4^ and PlantTFDB v 4.0^5^ (Jin et al., 2017). The search for the *GATA1* gene of *Momordica charantia* (bitter gourd) was conducted in the bitter gourd transcriptome available from NCBI (Urasaki et al., 2017). All alignments were performed using Clustal Omega at default settings ^6^. Phylogenetic trees were constructed in MEGA7.0 (Kumar et al., 2016). The neighbor-joining method (Saitou and Nei, 1987) of phylogenetic reconstruction was used with the Poisson model (Zuckerkandl and Pauling, 1965) with rate uniformity among sites. The maximum likelihood method of phylogenetic reconstruction was used with the Whelan and Goldman + Freq. model (Whelan and Goldman, 2001) with the rate of variation across sites (+G parameter = 3.7181) (Yang, 1994) and with the proportion of invariable sites (+I) (Shoemaker and Fitch, 1989). This model was chosen from the list of models with the lowest Bayesian Information Criterion (BIC) scores. Models with the lowest BIC scores are considered to best describe the substitution pattern. The maximum likelihood tree inference options were used at default settings. Bootstrap tests with 1000 replicates were used.

### Fluorescence Protein Reporter Assay

For the localization of eGFP-H2B and mNeonGreen-H2B reporters, 7–10 mm long tips of transgenic hairy roots of squash were vacuum infiltrated with a fixative (McLean and Nakane, 1974) modified by Brian Lin (Tufts University, Boston, MA, United States): 1% paraformaldehyde, 5% DMSO, 0.1 M L-lysine, 0.01 M sodium-m-periodate in 0.02 M phosphate buffer (PB) pH 7.2 for eGFP and pH 8.0 for mNeonGreen, fixed for 1 h at RT and rinsed with 0.02 M PB pH 8.0. The root tips were sectioned with a vibrating-blade microtome as described previously (Ilina et al., 2018). Nuclei were counterstained for 30–50 min with 0.3 μg/ml DAPI. Longitudinal or cross sections (65 μm) of root tips were mounted in consecutive order onto microscope slides in a non-hardening antifade mountant CFMR2 (Citifluor, London, United Kingdom) for eGFP or in PB pH 8.0 supplemented with 0.1 M L-lysine for mNeonGreen under coverslips.

### Microscopy

All microscopy procedures, three-dimensional reconstructions, animations, and maximum intensity projections were performed as described previously (Kitaeva et al., 2016; Ilina et al., 2018). Examination and imaging of fluorescent protein patterns were performed under a LSM 780 upright confocal laser scanning microscope (ZEISS, Germany) equipped with a Plan-Apochromat 20×/0.8 numerical aperture DICII objective and a Plan-Apochromat 40×/1.3 numerical aperture DICIII oil immersion objective. Samples were imaged with a 488 nm excitation laser line and an emission spectrum of 490–525 nm for both eGFP or mNeonGreen. For DAPI-stained nuclei, the 405 nm excitation laser line and an emission spectrum of 412–464 nm were used. A multitrack (line by line) scan mode was applied. The ZEN 2.3pro software (ZEISS) was used for image processing. At least 14 roots were used for each reporter assay. The distance from the initial cell to the first cell in file labeled with eGFP or NeonGreen in nuclei was measured in the ZEN software (ZEISS) after acquisition. Statistical analysis and graphical visualization were performed in SigmaPlot 12.5 (Systat Software, United States) using one-way analysis of variance (ANOVA) on ranks (Kruskal–Wallis).

## Results

### Identification of the Putative Ortholog of Arabidopsis GATA23 in *Cucumis sativus* and *Cucurbita pepo* by Phylogenetic Analysis

**FIGURE 1:**
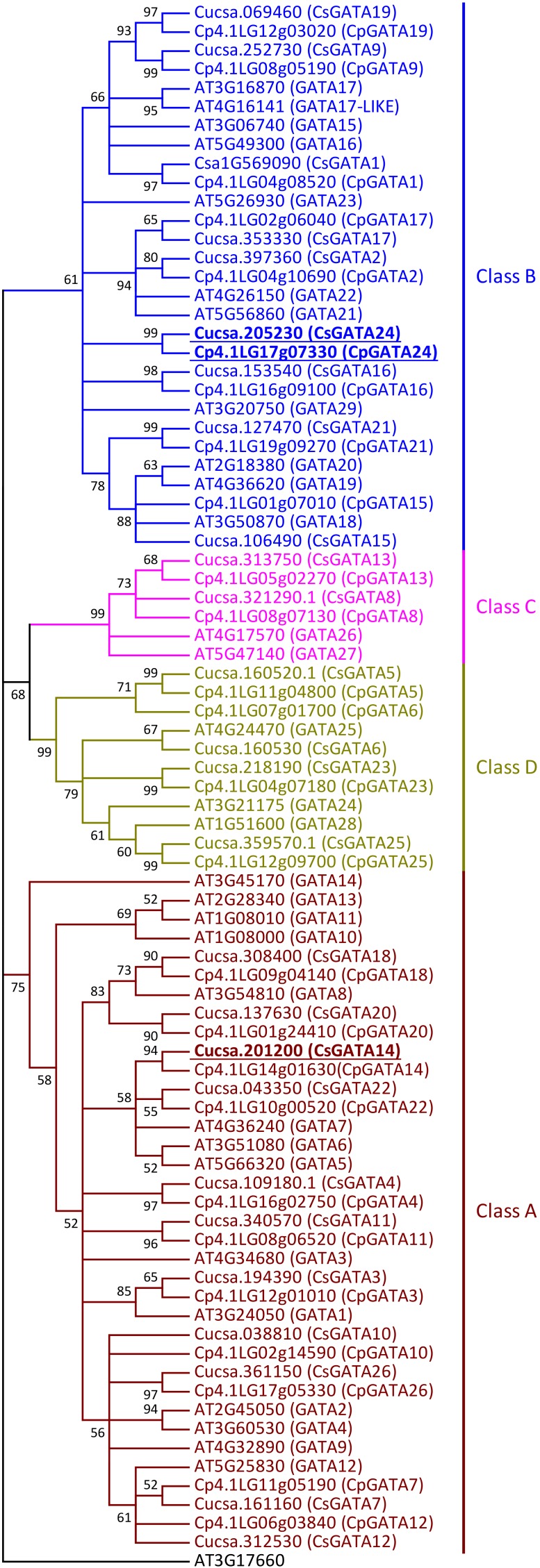
Neighbor-joining phylogenetic tree of the DNA-binding regions of GATA proteins from *Arabidopsis thaliana*, *Cucumis sativus*, and *Cucurbita pepo*. The phylogenetic tree was constructed based on a Clustal Omega alignment. Evolutionary analyses were carried out in MEGA7 software by the Neighbor-Joining method with 1000 bootstrap replicates using the Poisson model with rate uniformity among sites. All ambiguous positions were removed for each sequence pair. Bootstrap values are indicated by each node (≥50%). The amino acid sequence of the zinc finger domain from AT3G17660 protein was used as outgroup. The four different classes of GATA proteins are represented in different colors. Putative orthologs of *At*GATA23 in *C. sativus* and *C. pepo* are indicated (underlined bold). Gene ID prefixes: AT, *A. thaliana*; Cucsa, *C. sativus* Gy14 gene IDs on Phytozome; Csa, *C. sativus* Chinese Long v.2 gene IDs on Cucurbit Genomics Database; Cp, *C. pepo*.

Using BlastP searches in Phytozome, NCBI, Cucurbit Genomics Database, and PlantTFDB, 26 members of the GATA family of transcription factors were found both in cucumber (*C. sativus*) and squash (*C. pepo*). The 26 cucumber GATA transcription factors were named CsGATA1 to CsGATA26, according to their chromosomal positions, as was done for the *GATA* gene family of apple and soybean (Zhang et al., 2015; Chen et al., 2017), while squash GATAs were named according to their sequence similarity with cucumber GATA proteins (Supplementary Table S5). Phylogenetic analysis of GATA protein DNA-binding domains from cucumber and squash showed that, like in Arabidopsis (Reyes et al., 2004), cucurbit GATA transcription factors can be divided into four classes (Figure 1). The majority of *GATA* genes from cucumber and squash belong to classes A (11 genes per species) and B (eight genes). From 29 *GATA* genes in Arabidopsis, 14 and ten belong to the classes A and B, respectively. Two genes from cucumber and squash, respectively, belong to class C, compared to three genes in Arabidopsis. The number of genes from class D is equal both for Arabidopsis and cucurbits (four genes per species).

Like their homologs in Arabidopsis (Reyes et al., 2004), all squash and cucumber GATA proteins have only one DNA-binding domain (zinc-finger domain) with the core structure CX_2/4_CX_18/20_CX_2_C (Supplementary Figures S1, S2). Members of class A, B, and C have the structure CX_2_CX_18_CX_2_C (Supplementary Figure S1) with one exception: in cucurbits GATA16 protein (Class B), like in Arabidopsis GATA29 (Class B), the sequence of the zinc-finger domain is CX_4_CX_18_CX_2_C (Supplementary Figure S2). GATA proteins from class D show the domain structure CX_2_CX_20_CX_2_C (Supplementary Figure S1).

Since GATA23 belongs to class B (Behringer and Schwechheimer, 2015), the amino acid sequences of cucurbit GATAs of this class were analyzed in detail. B-GATAs with a HAN-domain and an LLM-domain have been described previously for Arabidopsis (Behringer et al., 2014; Behringer and Schwechheimer, 2015). Class B of cucurbit GATAs was divided into proteins with a HAN-domain and proteins with an LLM-domain (Supplementary Figures S2, S3). It was previously reported that cucumber contains two GATA genes encoding proteins with a complete HAN-domain, named HAN1 and HAN2 (Ding et al., 2015). Squash HAN-domain GATAs were given the same names according to their level of identity to cucumber GATA proteins. Cucurbit GATA16 proteins, like Arabidopsis GATA29, have a degenerate HAN-domain (Supplementary Figure S2).

Cucumber and squash have five GATA proteins with a complete LLM-domain: four of them have short amino acid sequences (GATA9, GATA17, GATA19, and GATA24), while one is a long protein (GATA2) (Supplementary Figure S3). For GATA1, which also belongs to the short proteins, the structure of the LLM-domain differs between cucurbit genera (Supplementary Figure S3). In the genus *Cucurbita*, it has the complete domain with the leucine-leucine-methionine sequence, as was also described for short Arabidopsis GATAs with a complete LLM-domain (Behringer and Schwechheimer, 2015). However, in *C. melo*, *C. lanatus*, *L. siceraria*, and *M. charantia*, the first leucine of the LLM-domain is substituted by serine. Interestingly, GATA1 from cucumber shows two substitutions in the LLM-domain: the first leucine is substituted by serine, like in melon, watermelon, calabash and bitter gourd, and methionine by isoleucine. Amino acid substitutions in cucumber GATA1 are similar to the substitutions in Arabidopsis GATA23 with regard to their positions. The degenerate LLM-domain from Arabidopsis GATA23 also contains substitutions of the first leucine and the methionine. However, the amino acid residues in these positions are different from those in cucumber GATA1: in Arabidopsis GATA23, the first leucine in the LLM-domain is substituted by cysteine and the methionine by leucine. Altogether, based on the phylogenetic analysis (Figure 1) and on the alignment of the GATA-domain and LLM-domain sequences in class B GATA proteins (Supplementary Figure S3), GATA1, GATA9, GATA17, GATA19, and GATA24 could represent putative orthologs of Arabidopsis GATA23.

### Cucurbit *GATA* Expression Levels Change in Response to Exogenous Auxin

The expression of Arabidopsis *GATA23* is upregulated by the auxin analog NAA at a concentration of 10 μM, leading to a maximal level of expression after 6 h (De Rybel et al., 2010). To identify orthologs of Arabidopsis *GATA23*, roots of cucumber and squash seedlings were treated with exogenous NAA, which can pass the plant plasma membrane by diffusion, while IAA requires uptake transporters (Marchant et al., 1999; Michniewicz et al., 2007). Expression levels of all members of the GATA family were compared in mock- and NAA-treated roots using reverse transcription – quantitative polymerase chain reaction (RT-qPCR).

**FIGURE 2:**
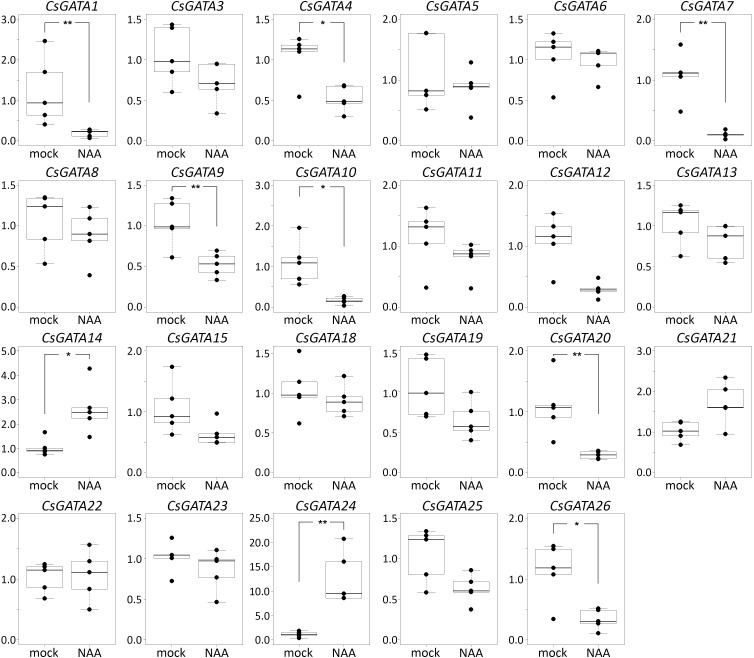
Relative transcript levels of *Cucumis sativus GATA* genes in response to NAA treatment. Four-day-old *C. sativus* seedlings were incubated with 10 μM NAA for 6 h. Reverse transcription-qPCR analysis was performed using RNA isolated from the first centimeter of the primary roots. Graphs were drawn using R software default code for boxplot function (Wilcoxon test, ^∗^*p* < 0.05, ^∗∗^*p* < 0.01). The *y* axis indicates the relative transcript level (2^−ΔΔCT^ method).

**FIGURE 3:**
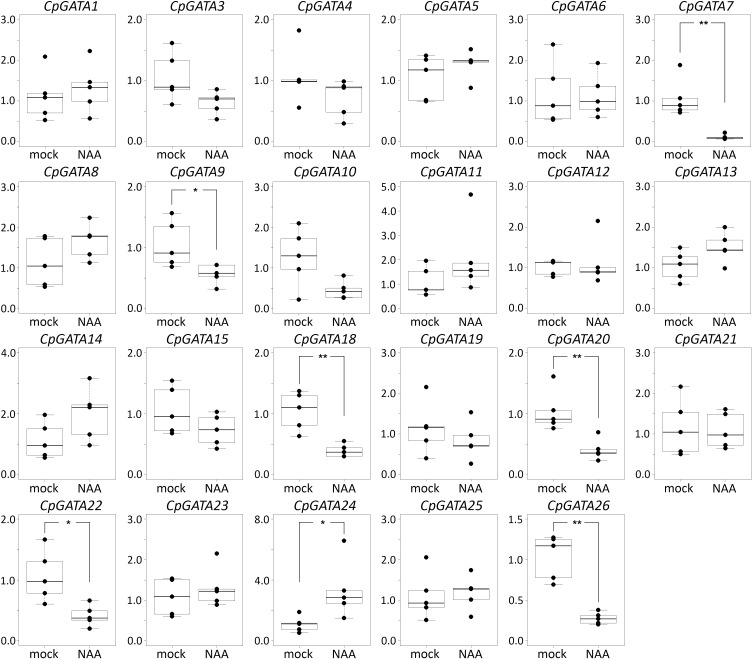
Relative transcript levels of *Cucurbita pepo GATA* genes in response to NAA treatment. Four-day-old *C. pepo* seedlings were incubated with 10 μM NAA for 6 h. RT-qPCR was performed using RNA isolated from the first centimeter of the primary roots. Graphs were drawn using R software default code for the boxplot function (Wilcoxon test, ^∗^*p* < 0.05, ^∗∗^*p* < 0.01). The y axis indicates the relative transcript level (2^−ΔΔCT^ method).

In cucumber and squash, the genes *GATA2*, *GATA16*, and *GATA17* were not expressed in roots, either with or without NAA treatment (Figure 2, 3). Most of the 26 *GATA* genes of both cucurbits were expressed in roots, but did not show significant changes in their expression levels in roots in response to exogenous NAA. This group included 15 cucumber *GATA* genes (*CsGATA3*, *CsGATA5*, *CsGATA6*, *CsGATA8*, *CsGATA11*, *CsGATA12*, *CsGATA13*, *CsGATA14*, *CsGATA15*, *CsGATA18*, *CsGATA19*, *CsGATA21*, *CsGATA22*, *CsGATA23*, and *CsGATA25*; Figure 2) and 16 squash genes (*CpGATA1*, *CpGATA3*, *CpGATA4*, *CpGATA5*, *CpGATA6*, *CpGATA8*, *CpGATA10*, *CpGATA11*, *CpGATA12*, *CpGATA13*, *CpGATA14*, *CpGATA15*, *CpGATA19*, *CpGATA21*, *CpGATA23*, and *CpGATA25*; Figure 3). For seven cucumber *GATA* genes (*CsGATA1*, *CsGATA4*, *CsGATA7*, *CsGATA9*, *CsGATA10*, *CsGATA20*, and *CsGATA26*; Figure 2) and six genes from squash (*CpGATA7*, *CpGATA9*, *CpGATA18*, *CpGATA20*, *CpGATA22*, and *CpGATA26*; Figure 3), NAA treatment led to a significant reduction of expression levels in roots. Only three *GATA* genes could be identified for which the expression levels increased in response to treatment with NAA: two from cucumber (*CsGATA14* and *CsGATA24*; Figure 2) and one from squash (*CpGATA24*; Figure 3). Expression levels of *CsGATA14* increased 2–3 fold. Transcript levels of *CsGATA24* were 6–20 fold higher in NAA-treated roots than in mock-treated roots, while relative expression levels of *CpGATA24* increased 2–6 fold.

Class B of cucurbit *GATA* genes consists of nine members (*GATA1*, *GATA2*, *GATA9*, *GATA15*, *GATA16*, *GATA17*, *GATA19*, *GATA21*, and *GATA24*; Figure 1). The expression of only one of these, *GATA24*, was upregulated in response to NAA in both cucumber and squash. The cucumber *GATA14* gene, the expression of which, like that of *GATA24*, was upregulated in response to NAA, belongs to class A. As mentioned above, cucurbits contain one class B *GATA* gene encoding a protein with a degenerate LLM domain, *GATA1*; the expression of this one was downregulated in response to NAA in cucumber, and not regulated in response to NAA in squash.

In summary, Arabidopsis *GATA23* belongs to class B, and the corresponding gene is upregulated by auxin. Only one cucurbit GATA shares these features, namely *GATA24* (gene ID in Phytozome Cucsa.205230 and in the Cucurbit Genomics Database Cp4.1LG17g07330; Supplementary Table S5). Phylogenetic (Figure 1) as well as expression analysis (Figure 2, 3) indicate that the *GATA24* genes of cucumber and squash represent putative functional orthologs of Arabidopsis *GATA23*.

### Identification of the Putative Ortholog of Arabidopsis MAKR4 in *Cucumis sativus* and *Cucurbita pepo* by Phylogenetic Analysis

**FIGURE 4:**
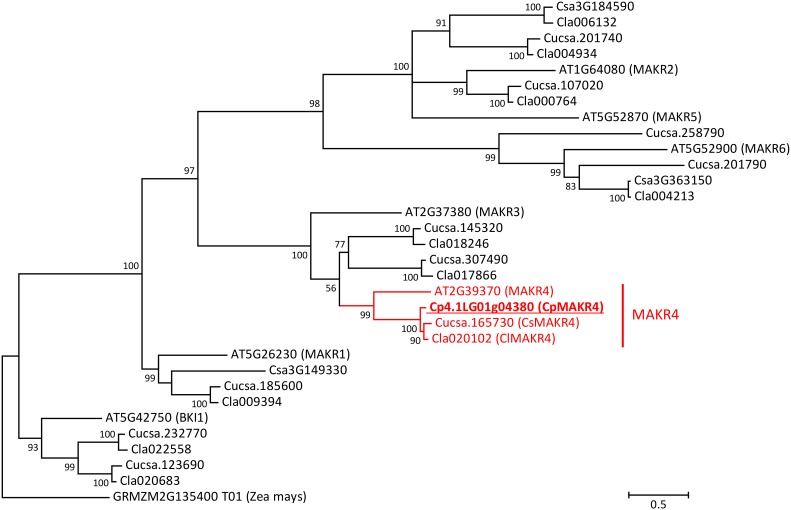
Phylogenetic tree of MAKR proteins from *A. thaliana*, *C. sativus*, *Citrullus lanatus*, and *C. pepo*. Amino acid sequence of putative ortholog of MAKR4 in *C. pepo* was used together with all known sequences of MAKR proteins from *A. thaliana*, *C. sativus*, and *C. lanatus*. The phylogenetic tree was constructed based on a Clustal Omega alignment. Evolutionary analyses were carried out in MEGA7 software using the Maximum Likelihood method with 1000 bootstrap replicates based on the gamma distributed (+G) Whelan and Goldman + Freq. model with invariant sites (+I). The amino acid sequence of the BKI1 protein (GRMZM2G135400_T01) from *Zea mays* was used as outgroup. The putative ortholog of *At*MAKR4 in *C. pepo* is indicated (underlined bold). Scale bar denotes 0.5 amino acid substitutions per site. Gene ID prefixes: AT, *A. thaliana*; Cucsa, *C. sativus* Gy14 on Phytozome; Csa, *C. sativus* Chinese Long v.2 on Cucurbit Genomics Database; Cla, *C. lanatus*; Cp, *C. pepo*.

The cucumber proteome contains 13 amino acid sequences of MEMBRANE-ASSOCIATED KINASE REGULATOR-like (MAKR) proteins (Figure 4). Six of these 13 proteins were annotated in Phytozome as members of MAKR family (gene IDs Cucsa.012770, Cucsa.165730, Cucsa.185600, Cucsa.201740, Cucsa.201790, and Cucsa.254170), while two of them were annotated as BKI1, a member of the MAKR protein family (gene IDs Cucsa.123690 and Cucsa.232770). Three truncated MAKR amino acid sequences, including two annotated as MAKRs, were found in Phytozome (gene IDs Cucsa.012770, Cucsa.108280, and Cucsa.254170); therefore, in this study they were replaced by the full-size protein sequences available from the Cucurbit Genomics Database (gene IDs Csa3G363150, Csa3G184590, and Csa3G149330). The watermelon (*C. lanatus*) genome encodes ten MAKR-like proteins (Figure 4). Two of these proteins were annotated in the Cucurbit Genomics Database as BKI1 (gene IDs Cla020683 and Cla022558), while eight of them were not annotated at all as MAKR-like proteins.

Phylogenetic analysis based on the MAKR protein family from Arabidopsis suggests that there is only one MAKR4 protein in cucurbits. The putative MAKR4 protein from squash (gene ID from the Cucurbit Genomics Database Cp4.1LG01g04380) showed 57% amino acid similarity and 45.3% identity with AtMAKR4 and therefore was assumed to be a putative ortholog (Figure 4). The *CpMAKR4* cDNA was cloned, sequenced, and deposited in NCBI GenBank under the accession number KY352352.

### *CpMAKR4* Expression Levels Differ in Their Response to Different Types of Auxin

**FIGURE 5:**
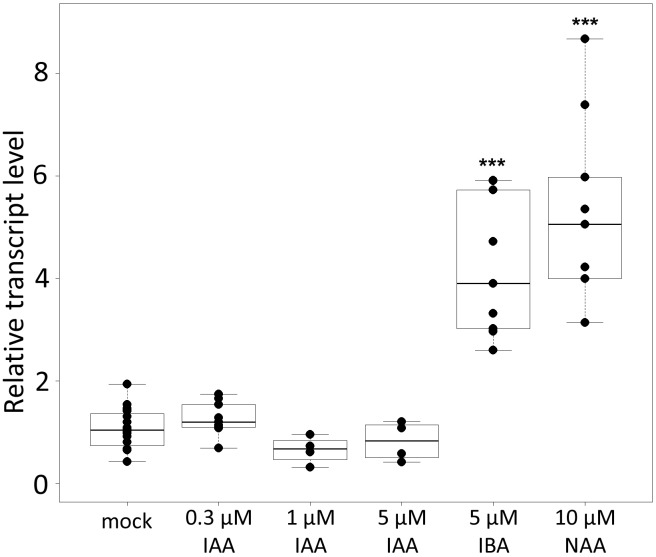
Relative transcript levels of *Cucurbita pepo MAKR4* in response to treatment with exogenous phytohormones. Four-day-old *C. pepo* seedlings were incubated for 6 h with 0.3, 1, 5 μM IAA, 5 μM IBA, or 10 μM NAA, respectively. RT-qPCR analysis was performed using RNA isolated from the first centimeter of the primary roots. Graphs were drawn using R software default code for the boxplot function (Wilcoxon test, ^∗∗∗^*p* < 0.001). The *y* axis indicates the relative transcript level (2^−ΔΔCT^ method).

To confirm that *CpMAKR4* represents a putative functional ortholog of Arabidopsis *MAKR4*, expression levels of *CpMAKR4* were analyzed in squash roots after treatment with different auxins (Figure 5). Arabidopsis *MAKR4* expression has been reported to be upregulated in response to IAA and IBA (Xuan et al., 2015). RT-qPCR analysis showed that transcript levels of *CpMAKR4* did not change in response to treatment with 0.3, 1, or 5 μM IAA for 6 h. However, expression levels increased 3–4 fold in response to treatment with 5 μM IBA, and 3–5 fold in response to 10 μM NAA.

### *CpGATA24* and *CpMAKR4* Expression Patterns in *Cucurbita pepo* Root

**FIGURE 6:**
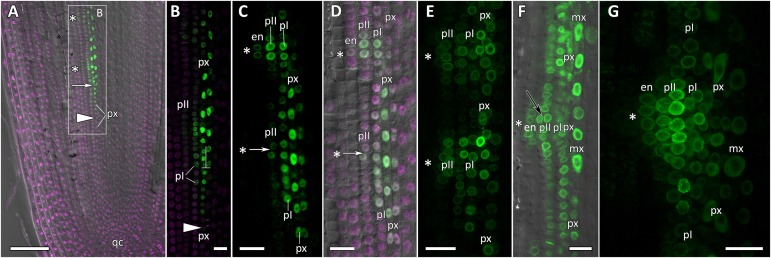
Localization of *CpGATA24* expression along the longitudinal axis of *Cucurbita pepo* root tips. Confocal laser scanning microscopy of longitudinal vibratome sections. Green channel – fluorescence of mNeonGreen-H2B, magenta channel – DNA in nuclei stained with DAPI, gray channel – differential interference contrast. **(A)** An overview and **(B)** close-up of the parental root meristem shows the acropetal sequence of *GATA24* promoter activity in protoxylem and pericycle. *GATA24* expression arises first in the protoxylem at a distance of 275 μm from the initial cells (arrowhead), before the formative T-division (arrow). **(C,D)** The establishment of *GATA24* activity in xylem, pericycle layers and endodermis. Founder cell specification in the outer pericycle (white arrows). Two endodermal cells with local *GATA24* activity can be seen in the upper developing primordium. **(E,F)** Asterisks indicate the position of young lateral root primordia after the first anticlinal and periclinal (black arrow) divisions in the pericycle. *GATA24* transcription can be seen in the protoxylem between primordia. **(G)** Lateral root primordium within the parental root meristem at a distance of 500–600 μm from the QC. **(A–D,F)** Single optical sections. Maximum intensity projection of *z*-series: **(E)** of 26 optical sections, 27 μm in depth; **(G)** of 47 optical sections, 50 μm in depth. Asterisks indicate developing lateral root primordia; en, endodermis; mx, metaxylem; pI, inner pericycle layer; pII, outer pericycle layer; px, protoxylem; qc, quiescent centre. Scale bars denote 100 μm in **(A)**, and 20 μm in **(B–G)**.

**FIGURE 7:**
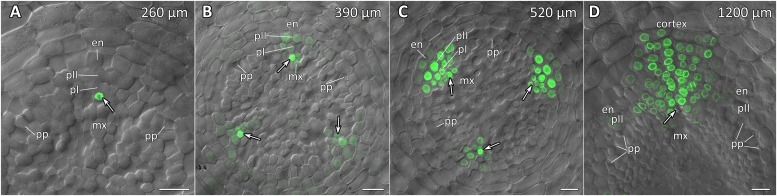
Radial pattern of *CpGATA24* expression along the longitudinal axis of *Cucurbita pepo* root tip. Confocal laser scanning microscopy of vibratome cross-sections. Overlays of single optical sections with differential interference contrast and maximum intensity projections of a *z*-series of mNeonGreen-H2B fluorescence in the green channel (32 optical sections, 36 μm in depth). Distances from initial cells are shown in the upper right corner of each panel. **(A)** Expression of *GATA24* in the protoxylem (arrow). **(B)** In both pericycle layers, expression of *GATA24* begins in the founder cells (arrows point at protoxylem cells expressing *GATA24*). **(C)** Before periclinal divisions in the pericycle (stage I of lateral root primordium formation), expression of *GATA24* is induced in 2–3 files of the endodermis, too. **(D)** Lateral root primordium at a distance of 1200 μm from the initial cells. Protoxylem undergoes terminal differentiation (arrow). Arrows indicate protoxylem; en, endodermis; mx, metaxylem; pI, inner pericycle layer; pII, outer pericycle layer; pp, protophloem. Scale bars denote 20 μm.

**FIGURE 8:**
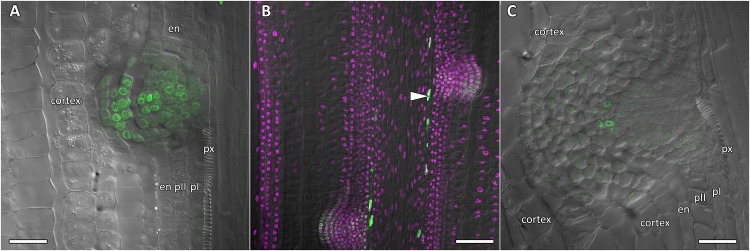
Localization of *CpGATA24* expression in lateral root primordia of *Cucurbita pepo* root. Confocal laser scanning microscopy of longitudinal vibratome sections. Green channel – fluorescence of mNeonGreen-H2B, magenta channel – DNA in nuclei stained with DAPI, gray channel – differential interference contrast. **(A)** Lateral root primordium at a distance of 1000 μm from the QC. **(B)** *CpGATA24* expression is remained in metaxylem cells (arrowhead) and lateral root primordia at a distance of 1500 μm from the QC. **(C)** *CpGATA24* expression has disappeared in lateral root primordia at a distance of 2000 μm from the QC. en, endodermis; pI, inner pericycle layer; pII, outer pericycle layer; px, protoxylem. Scale bars denote 50 μm in **(A,C)**, and 100 μm in **(B)**.

**FIGURE 9:**
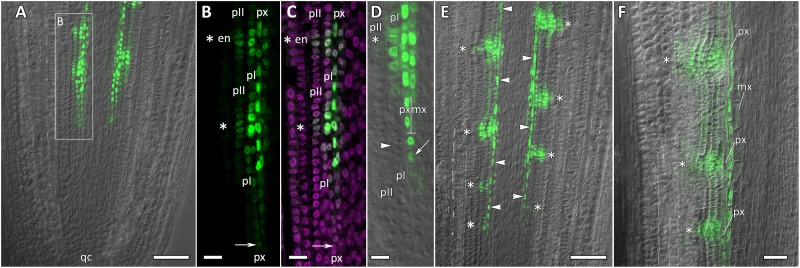
Localization of *CpMAKR4* expression along the longitudinal axis of *Cucurbita pepo* root tips. Confocal laser scanning microscopy of longitudinal vibratome sections. Green channel – fluorescence of eGFP-H2B, magenta channel – DNA in cell nuclei stained with DAPI, gray channel – differential interference contrast. **(A)** An overview and **(B,C)** close-up of the parental root meristem shows the acropetal sequence of *CpMAKR4* promoter activity in protoxylem and pericycle. *CpMAKR4* expression arises first in the protoxylem at a distance of 330 μm from the initial cells (arrows). **(D)** *CpMAKR4* expression takes place just before the last formative T-division in the protoxylem. An arrow points at the first cell expressing *CpMARK4* in the protoxylem cell file. Two putative founder cells in the inner pericycle layer are labeled with an arrowhead. The first anticlinal division (metaphase) in an inner pericycle cell expressing *CpMAKR4* is visible (asterisk). **(E)** Young lateral root primordia (asterisks) and protoxylem cells between primordia (arrowheads) expressing *CpMAKR4*. **(F)** *CpMAKR4* expression is remained in protoxylem cells and lateral root primordia at a distance of 1500 μm from the initials. Asterisks indicate developing lateral root primordia; en, endodermis; mx, metaxylem; pI, inner pericycle layer; pII, outer pericycle layer; px, protoxylem; qc, quiescent centre. Scale bars denote 100 μm in **(A,E)**, 20 μm in **(B,C)**, 10 μm in **(D)**, 50 μm in **(F)**.

**FIGURE 10:**
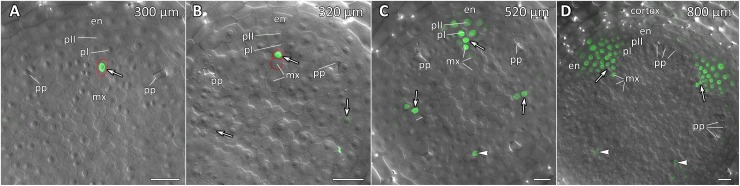
Radial pattern of *CpMAKR4* expression along the longitudinal axis of *Cucurbita pepo* root tip. Confocal laser scanning microscopy of vibratome cross-sections. Overlays of single optical sections with differential interference contrast and maximum intensity projections of a *z*-series of eGFP-H2B fluorescence in the green channel (16 optical sections, 15 μm in depth). Distances from initial cells are given in the right upper corner of each panel. **(A)** Expression of *CpMAKR4* in the protoxylem (arrow, cell outlined in red) before the T-division. **(B)** Two cell files (outlined in red) are formed at a xylem pole. Expression of *CpMAKR4* in the protoxylem is visible. **(C)** Expression of *CpMAKR4* is induced in the founder cells of the inner and outer pericycle layer, also in metaxylem cells opposite young lateral root primordia. **(D)** Lateral root primordia at a distance of 800 μm from the initial cells. Expression of *CpMAKR4* takes place in 3–4 files of the endodermis as well. Protoxylem and metaxylem (arrowheads) cells between primordia show *CpMAKR4* transcription. Arrows indicate protoxylem; en, endodermis; mx, metaxylem; pI, inner pericycle layer; pII, outer pericycle layer; pp, protophloem. Scale bars denote 20 μm.

Transgenic squash hairy roots harboring promoter fusion constructs *CpGATA24::mNeonGreen-H2B* or *CpMAKR4::eGFP-H2B* were used to investigate the expression patterns and involvement in lateral root primordium initiation of the corresponding genes. To visualize gene expression patterns, a series of longitudinal (Figure 6, 8, 9) and cross (Figure 7, 10) root sections were analyzed.

### Expression of *CpGATA24* Is Induced in the Protoxylem Before Founder Cell Specification in the Pericycle

Expression of *CpGATA24* began in the protoxylem at an average distance of 265 μm from the initial cell (Figure 6A,B, 7A and Supplementary Figure S4). This event occurred 50–60 μm before the periclinal T-division in a file of protoxylem cells (Figure 6B, 7A). Such a division leads to the formation of two xylem files. The outer file is usually designated as protoxylem, and the inner one as peripheral metaxylem (Figure 6B, 7A). Throughout a file, *CpGATA24* expression was maintained in all cells of the protoxylem and peripheral metaxylem (Figure 6, 7, 8) until a point at about a distance of 1500 μm from the initial cells where terminal differentiation of the protoxylem cells commenced. In the pericycle, expression of *CpGATA24* began at a distance of approx. 300 μm from the initial cells, before the first anticlinal divisions (Figure 6B–D). This occurred in the cells of the inner pericycle layer approximately opposite to the T-division in the protoxylem. Expression of *CpGATA24* in the cells of the inner pericycle layer was triggered simultaneously in several (6–8) cells (Figure 6C,D). In the outer layer of the pericycle, expression was induced a bit later and only in the putative founder cells (Figure 6C,D, 7C). Upon completion of the first anticlinal formative divisions in the pericycle, expression of *CpGATA24* was extended to the endodermal cells involved in the formation of the lateral root primordium (Figure 6C,D, 7B,C). When the first periclinal divisions took place in a primordium (Figure 6F,G), *CpGATA24* expression was extended also to the files of stelar parenchyma surrounding the peripheral metaxylem (Figure 7B–D). The pattern of *CpGATA24* expression in the cells of the inner pericycle layer was also maintained between the developing primordia until a distance of approx. 600 μm from the initial (Figure 6E–G). Cells of 2–3 inner cortical layers opposite the primordium also expressed *CpGATA24* (Figure 7D, 8A). At a distance of approx. 1 mm from the initial cells, expression of *CpGATA24* was maintained in all primordium cells (Figure 8A). *CpGATA24* expression was maintained in all stelar parenchyma cells adjacent to the protoxylem until a distance of 1.5–2 mm from the initial. Then, the number of cells expressing *CpGATA24* in primordia decreased gradually (Figure 8B). The last *CpGATA24* expressing cells were found in the zone of initial cells of lateral root primordia at a distance of approx. 2–2.5 mm from the root tip (Figure 8C).

Thus, the expression of *CpGATA24* is directly correlated to the determination of the site of lateral root initiation at a xylem pole. Protoxylem cells appear to play a crucial role in the positioning of the primordium. *CpGATA24* expression extends from the protoxylem file to the founder cells in the pericycle, immediately after the completion of the last periclinal formative division in the protoxylem.

### Expression of *CpMAKR4* Is Induced in the Protoxylem Before the First Formative Division in Pericycle

*CpMAKR4* expression began in the protoxylem at an average distance of 330 μm from the initial cell (Figure 9A–D, 10A and Supplementary Figure S4). This occurred just before the periclinal T-division in the protoxylem, which leads to a split of this cell file to the protoxylem and peripheral metaxylem files (Figure 9B,D, 10A,B). *CpMAKR4* expression was maintained in the protoxylem and peripheral metaxylem until a distance of approx. 2–2.5 mm (Figure 9F). In the inner layer of the pericycle, the expression of this gene was triggered a bit later than in protoxylem (Figure 9B–D). In Figure 9D, the site of lateral root primordium initiation is shown with an asterisk: one of the two sister cells of the inner pericycle layer has already divided, and the second (basal) is in the metaphase. All three cells show activity of the *CpMAKR4* promoter. The cells of the outer layer of the pericycle, already involved in the initiation of the lateral root primordium, have begun to express *CpMAKR4* in pairs of cells immediately before the first formative anticlinal divisions (Figure 9B,C). As the primordium developed, proceeding to involve the endodermis and several layers of the inner cortex, *CpMAKR4* expression extended to all cells of the primordium (Figure 9E,F, 10C,D). The decrease in its expression level in primordium cells was complete at a distance of 3 mm from the root tip.

Thus, *CpMAKR4* expression begins in the protoxylem, shortly before the first formative divisions in the pericycle, and is likely to be connected with the determination of the site of lateral root initiation at a xylem pole. *CpMAKR4* expression is triggered directly before the anticlinal divisions of the pericycle cells, which initiate the formation of a lateral root primordium. The expression of this gene is maintained for the entire subsequent development of the primordium, before it emerges from the parental root.

## Discussion

The first problem we sought to address in this study was the identification of orthologs of Arabidopsis GATA23 and MAKR4 in cucurbits. The number of *GATA* genes identified in cucumber and squash genomes was 26 for each species, which is less than in the Arabidopsis genome (Reyes et al., 2004). Therefore, it could be assumed that some gene family members in Arabidopsis did not have an ortholog in cucumber and/or squash. Using phylogenetic analysis, cucurbit GATA transcription factors were divided into four classes according to their DNA-binding domain structure like in Arabidopsis and rice (Reyes et al., 2004). All squash and cucumber GATA proteins have the CX_2/4_CX_18/20_CX_2_C structure of the zinc-finger domain, which is typical for GATA proteins from the entire plant kingdom (Reyes et al., 2004). Phylogenetic analysis did not allow the unambiguous identification of the ortholog of Arabidopsis GATA23 in cucurbits. Another criterion had to be added, namely that the expression of a putative functional ortholog of Arabidopsis *GATA23* had to be upregulated in roots by exogenous application of auxin (De Rybel et al., 2010). Only one gene from cucurbits, *GATA24*, fulfilled the phylogenetic and transcriptional criteria and was therefore proposed as the putative functional ortholog of Arabidopsis *GATA23*.

Phylogenetic search for a putative ortholog of Arabidopsis *MAKR4* in the squash genome was easy in that there is only one *MAKR4* gene in squash despite the whole genome duplication event postulated for the genus *Cucurbita* (Montero-Pau et al., 2018). Here, cucumber was not included in the search. Transcriptional analyses on the effects of auxin were performed with 10 μM of the synthetic auxin NAA for GATA genes. For MAKR, however, three effects of different auxins – the synthetic NAA and the natural IAA and IBA – were compared, with the result that only NAA and IBA had an effect. Thus, in cucurbits IBA must be converted to IAA in the root cap, as was previously reported for Arabidopsis (De Rybel et al., 2012; Xuan et al., 2015). The lack of induction of *CpMAKR4* by the natural auxin IAA in spite of the effect of the synthetic auxin NAA could be explained by the fact that, as was shown for Arabidopsis, while NAA can pass the plasma membrane by diffusion, IAA requires active uptake systems (Marchant et al., 1999; Michniewicz et al., 2007).

Arabidopsis *GATA23* and *MAKR4* function in a cell-autonomous manner (De Rybel et al., 2010; Xuan et al., 2015); thus, it is very likely that their homologs in Cucurbitaceae also act cell-autonomously. Therefore, the analysis of their promoter activities should indicate the distribution of the corresponding proteins.

The expression pattern of *GATA23* in Arabidopsis has been analyzed in a few studies (De Rybel et al., 2010; Xuan et al., 2015; Murphy et al., 2016). The authors concluded that *GATA23* expression is induced before the first asymmetric division and synchronous nuclear migration in two pericycle cells, and that this expression is only maintained up to and including stage II. That is, *GATA23* expression finishes with the first periclinal divisions (De Rybel et al., 2010). According to Murphy et al. (2016), *GATA23* expression is triggered in the pericycle at xylem poles within the elongation zone. Xuan et al. (2015) also convincingly showed the induction of *GATA23* expression in two pericycle cells before the asymmetric formative division. In addition, the cellular auxin response maxima (as evidenced by *DR5* promoter activity) also appear in two pericycle cells just before the nuclear migration (De Rybel et al., 2010). *GATA23* expression is controlled by the activity of the Aux/IAA28-dependent signaling module in the basal part of the apical root meristem (De Rybel et al., 2010), where the priming of founder cells occurs. Altogether, based on the expression pattern of *GATA23*, it was concluded that specification of founder cells takes place in the pericycle above the elongation zone (De Rybel et al., 2010; Lavenus et al., 2013), and that GATA23 and a small signaling peptide, GLV6, may be involved in the nuclear migration mechanism in polarized founder cells (De Rybel et al., 2010; Fernandez et al., 2015).

During the initiation of lateral root primordia within the elongation zone, nuclear migration is typically observed in two adjacent pericycle cells prior to the asymmetric division (Casero et al., 1993; Demchenko and Demchenko, 1996; Casimiro et al., 2003; De Rybel et al., 2010). The coordinated, synchronous nuclear migration of two neighboring pericycle nuclei in Arabidopsis roots depends on Aux/IAA28 and ARF-dependent signaling and is an absolute prerequisite for primordium initiation (De Rybel et al., 2010). In contrast, in *C. pepo* roots, where the initiation of lateral root primordia takes place in the parental root meristem, the cells of different pericycle files are short (meristematic) and their nuclei are already positioned near the center of future primordia. In this case, nuclear migration is unnecessary (Ilina et al., 2017, 2018). Most likely, *CpGATA24*, the putative functional ortholog of *GATA23*, does not target genes required for coordinated nuclear migration in the pericycle (Bone and Starr, 2016).

In the current study, we show that the pattern of expression of *CpGATA24* is much broader than that of *GATA23*. The onset of *CpGATA24* expression is associated with the protoxylem, and only expands to individual pericycle cells (the founder cells) before the anticlinal formative divisions that lead to lateral root initiation (Figure 6, 7). In addition, *CpGATA24* expression is maintained in all primordial cells up to and including stage V, gradually decreasing in the later stages (Figure 8). Comparative analysis of cellular auxin response maxima (Ilina et al., 2018) and *CpGATA24* expression shows that in *C. pepo*, unlike in Arabidopsis, they do not coincide. At a distance of approx. 390 μm from the initials, an auxin response maximum is present in all metaxylem files, but *CpGATA24* is only expressed in the protoxylem and the peripheral metaxylem. At larger distances from the root tip, the *CpGATA24* expression pattern is wider than the cellular auxin response maximum. It is important to note that in the protoxylem as well as in adjacent cell files of the stelar parenchyma, *CpGATA24* expression is constant in all cells of a given file up to a distance of approx. 1500 μm from the initials, in contrast with the expression of *GATA23* in Arabidopsis which is only induced in pre-branch sites and maintained during lateral root primordia formation (De Rybel et al., 2010). This might indicate the presence of an additional transcriptional activator of *CpGATA24* beyond auxin signal transduction. Based on the expression pattern of *CpGATA24* in the central cylinder, this putative trigger would likely move through the protoxylem file from the basal parts of the root down to the apical meristem.

In Arabidopsis, MAKR4 converts the prebranch sites into a regular spacing of lateral roots (Xuan et al., 2015). In the sequence of events leading to founder cell specification, induction of *MAKR4* expression usually takes place after induction of *GATA23* expression. The expression of the squash *CpMAKR4* gene, the putative functional ortholog of *MAKR4*, is activated in the protoxylem a little bit later than that of *CpGATA24* (Supplementary Figure S4). Interestingly, both of these events occur before the last periclinal T-division, which leads to the formation of the cell files of proto- and metaxylem (Figure 6A, 9D, 10A,B). In squash, *CpMAKR4* and *CpGATA24* expression is maintained in all protoxylem cells, while in Arabidopsis roots, *MAKR4* is only expressed in the protoxylem of the parental root in the basal part of the lateral root meristem (Xuan et al., 2015).

Above the elongation zone in Arabidopsis, MAKR4 is located at the plasma membrane on the border of two adjacent founder cells in the pericycle prior to the synchronous nuclear migration (Xuan et al., 2015). After completion of the first anticlinal division, it is localized throughout the plasma membrane of sister cells. During further primordium development, *AtMAKR4* is expressed in overlaying cell layers of early-stage lateral root primordia. Also, in the pericycle of squash roots, *CpMAKR4* expression is induced directly before the first anticlinal division (Figure 9B–D), and this always happens in two adjacent founder cells. Subsequently, *CpMAKR4* expression is maintained in primordia up to stage V (Figure 9E,F). This is consistent with a direct involvement of *CpMAKR4* in the development of lateral root primordia before the formation of their own initial cells in the quiescent center of the emergent lateral root meristem, and is also consistent with the defect in lateral root emergence observed in a *MAKR4* knock-down line (Xuan et al., 2015).

In Arabidopsis, it was proposed that periodic fluctuations in auxin distribution or responsiveness determine the longitudinal spacing of lateral root initiations (De Smet et al., 2007). Because of the failure of exogenously applied IAA to affect the frequency of pulses in the oscillation zone, it was concluded that the pace of oscillation is set by an endogenously regulated clock with a stable, auxin-independent periodicity (Moreno-Risueno et al., 2010; Van Norman et al., 2013), suggesting that the pathway determining prebranch site formation is largely independent of the pathway leading to lateral root formation. In Arabidopsis, the specific induction of expression of *GATA23* and *MAKR4* in founder cells in the pericycle cell files can only be explained by the oscillation of a trigger. Yet, in squash, lateral root initiation takes place at a distance of 250–300 μm from the initial cells, indicating that prebranch site formation takes place even closer to the root tip. Thus, it is unlikely that in cucurbit roots there is enough space for the formation of such auxin oscillations as were described for Arabidopsis basal meristem. However, the expression of *CpGATA24* and *CpMAKR4* in cell files of the stelar parenchyma argues for the involvement of a second regulator that on the one hand is independent of auxin, and on the other hand likely moves from the basal part of the root toward the meristem. Since the trigger of *CpGATA24* and *CpMAKR4* expression always extends centrifugally from the protoxylem, speculation about the movement of auxin as a trigger from dying root cap cells in Arabidopsis (Möller et al., 2017) cannot apply to Cucurbitaceae.

Further identification of the target genes of the transcription factor GATA23 and of its orthologs in other plants, as well as the elucidation of the actual role of MAKR4 in the signaling cascade that leads to prebranch sites formation, will shed light on the mechanisms of formation of local competence for lateral root initiation in pericycle cells. Expansion of our knowledge in this area will also lead to the identification of mechanisms of systemic regulation of branching, which is necessary for the development of targeted breeding strategies in agriculture.

## Author Contributions

KND and KP planned and designed the research. ASK, ELI, KND, EDG, and VAP performed most experiments and analyzed data. ASK performed phylogeny and bioinformatics. KND and ELI carried out the design of all vectors. KND performed the microscopy. KND, KP, ASK, and ELI wrote the manuscript. All authors contributed to the final version.

## Conflict of Interest Statement

The authors declare that the research was conducted in the absence of any commercial or financial relationships that could be construed as a potential conflict of interest.

## Footnotes

1 www.arabidopsis.org

2 cucurbitgenomics.org

3 phytozome.jgi.doe.gov

4 www.ncbi.nlm.nih.gov

5 planttfdb.cbi.pku.edu.cn

6 www.ebi.ac.uk/Tools/msa/clustalo
