# Supplementary material for: Lateral Root Initiation in the Parental Root Meristem of Cucurbits: Old Players in a New Position
Source: Front Plant Sci. 2019 Apr 10;10:365. doi: 10.3389/fpls.2019.00365 (PMC6499211; doi:10.3389/fpls.2019.00365)
Supplement: Supplementary file 1 [file Data_Sheet_1.pdf]

Supplementary Material

Lateral Root Initiation in the Parental Root Meristem of Cucurbits: Old Players in a New Position

Alexey S. Kiryushkin, Elena L. Ilina, Elizaveta D. Guseva, Vera A. Puchkova, Katharina Pawlowski* and Kirill N. Demchenko*

*Correspondence:
Kirill N. Demchenko demchenko@binran.ru
Katharina Pawlowski katharina.pawlowski@su.se

The following Supplementary Material is available for this article:

Supplementary Table S1. Construction of entry vectors.

Name of insert	Template	Source of template	Name of vector used	Source of vector used	Resulting Entry vector
mNeonGreen-H2B-C6	Allele Biotechnology plasmid #H2B-213	Shaner et al. (2013)	pUC18-entry8	Hornung et al. (2005)	*mNeonGreen-H2B*- pUC18-entry8 vector
eGFP-H2B	Addgene plasmid #56436	gift from Michael Davidson	pUC18-entry8	Hornung et al. (2005)	*eGFP-H2B* - pUC18-entry8 vector
A. thaliana act2 terminator	Addgene plasmid pICH44300	gift from Sylvestre Marillonnet & Nicola Patron	373_pENTRattR2 attL3	Thermo Fisher Scientific	373_pENTRattR2attL3-TerAct

Supplementary Table S2. Construction of binary vectors.

Binary vector	Destination vector	Promoter in Entry vector	Reporter in Entry vector	Terminator in Entry vector
242_pKGW-RR-MGW-pCpGATA24::mNeonGreen-H2B	242 pKGW-RR-MGW	pCpGATA24-369_pENTRattL4attR1_BSAI	*mNeonGreen-H2B*-pUC18-entry8	373_pENTRattR2attL3-TermAct
242_pKGW-RR-MGW-pCpMAKR4::eGFP-H2B	242_pKGW-RR-MGW	pCpMAKR4-369_pENTRattL4attR1_BSAI	*eGFP-H2B* - pUC18-entry8 vector	373_pENTRattR2attL3-TermAct

Supplementary Table S3. List of PCR primers used for amplification of promoters/coding regions in this study.

Name	Restriction enzyme/att site	Sequence 5`-3`
pCpMAKR4 FOR	*SmaI*	a**CCCGGG**cgaaactccgcctccaagagc
pCpMAKR4_Rev	*SmaI*	a**CCCGGG**gaaactatgctttatcaggtggttcaagg
pCpGATA24_For1	*SmaI*	a**CCCGGG**cgtaacgagctaaaacaggcaatatctact
pCpGATA24_Rev1	*SmaI*	a**CCCGGG**cagagagatcgggatggttgttga
H2B FOR	*KpnI*	aaa**GGTACC**atgccagagccagcgaagtc
eGFP-H2B REV	*NotI*	aa**GCGGCCGC**gtacagctcgtccatgccgag
NeonGreen-H2B REV	*NotI*	aa**GCGGCCG**Cttacttgtacagctcgtccatgcc
TermAct FOR	AttBr2	GGGG**ACAGCTTTCTTGTACAAAGTGGCC**TAACTCTGTGGT CTCAGCTTGCTCTCA

TermAct REV	AttB3	GGGG**<u>ACAACTTTGTATAATAAAGTTGA</u>**CTCAAGCGAAATGGTGCGATCT

Restriction enzyme/att sites in adaptors are underlined and given in **BOLD** print.

Supplementalry Table S4. List of qPCR primers used in this study.

Name	Sequence 5`–3`	Amplicon size, bp	Type of detection
CsEF1a FOR	ATGGGTAAGGAGAAGGTTCACATTAACATT	241	SYBR
CsEF1a REV	CGAACTTCCACAAAGCAATATCAATT		
CsGATA1 FOR	AATATCCACCAGAGGAACGAACAGAC	185	
CsGATA1 REV	TTCTCAATGACCCACACTCCCCT		
CsGATA2 FOR	CACAATGATTCGGTGAAGTGGTCTTCT	145	
CsGATA2 REV	ACGGTGACGGTGAGTTGTTGAGATC		
CsGATA3 FOR	ACGACGCCGTTCCACCCTTT	299	
CsGATA3 REV	GATTGAAGTGCTTTCCAGAACCGAA		
CsGATA4 FOR	CCATTGGGAATTAGCCATGAAATCT	261	
CsGATA4 REV	GAATCCTCCTCTCCGACGACCG		
CsGATA5 FOR	ATGATGGCGGATGACGACGG	274	
CsGATA5 REV	GGTACTTCCATTGTAGGTACATCTGCTGG		
CsGATA6 FOR	GGGTCCGACTTGGCTGTTCAG	218	
CsGATA6 REV	AGGTTGACTACTCCGTCCAGGCAA		
CsGATA7 FOR	TCCTCCTTCTTTGAAGATATTAGTGGCTC	246	
CsGATA7 REV	CGGTTTGAAAATTGCTGCTGC		
CsGATA8 FOR	CGAACAACAGTCTTACTTTTCTGGATCTTC	284	
CsGATA8 REV	CAGTAGAGTGCTTCCTCCCAATTCC		
CsGATA9 FOR	TCGTCGGAGGAGAGTCAAGTGAAT	252	
CsGATA9 REV	AAGCCTTTGTTTCAAACTATCTCTGAACTT		
CsGATA10 FOR	TTGATAAGGATTTTCCAGCAATTCCATT	249	
CsGATA10 REV	CCATCGTCCCTTCCGTTTTCTC		
CsGATA11 FOR	CACCCTCCCTCTTCCACCACC	223	
CsGATA11 REV	AGCAGGTTCAGTTGACGGAGACG		
CsGATA12 FOR	TTCCCGATTATCCGTACTCTCTCCC	110	
CsGATA12 REV	TTTCTTTCTTCTTCGTGGTTTTCTTGGC		
CsGATA13 FOR	GCCCCTATTTCAAGGAGAACTTAACTTC	213	
CsGATA13 REV	CATTGGCATGGGAAAGAAAAGGTC		
CsGATA14 FOR	TTCCTTCAAGACCAAACCCCTGAT	280	
CsGATA14 REV	CCTCCACCAGTTGTTCCTCCGAG		
CsGATA15 FOR	CACCCAATGTTCAACGGAAGTTATACAA	206	
CsGATA15 REV	CATCTTGTGAAATCATGGACGAGAGTG		
CsGATA16 FOR	CTTCATCCAAGTTCATACCCTAATACTCAT	225	
CsGATA16 REV	GACATCATTGGTGGCTGAAACTCG		
CsGATA17 FOR	GCAAAAGATGATGATAAACACAAACCATAA	154	
CsGATA17 REV	CGCTTCCCATTTTCCACCGTTT		
CsGATA18 FOR	CGTAGTCCTGAACCAACCGTCG	261	
CsGATA18 REV	GATCTTCTGTGTTCCCGACCCAG		
CsGATA19 FOR	ATGCTGGAAAGACATCGCCGA	234	
CsGATA19 REV	CACCGCCATTGTTGTTTCTGTTG		
CsGATA20 FOR	AGCCTTTCCTCCCATTTGGTCTG	282	
CsGATA20 REV	GCTGGAGCTGCTGCTGCTACTG		
CsGATA21 FOR	GTTCTTGTAATGGATTTTACGGGCAA	246	SYBR
CsGATA21 REV	GGGTGGTTTTGGAGGAATGAGG		
CsGATA22 FOR	TCAGATGAAGAACAGCAAGAAGATGAAGA	310	
CsGATA22 REV	GTTTTGAAGGAAATGGGGTGTTGAG		
CsGATA23 FOR	ACGGCGGGGAAGAGTCCATAG	277	
CsGATA23 REV	GCAGGAATACCAGAAGGAATTTCATATCC		
CsGATA24 FOR	GAAGAAAATGGGAGGATCAGGAGG	164	
CsGATA24 REV	ATAAAGCCATAAGAAGCAACGCTGC		
CsGATA25 FOR	TATATGAGTAATGGGAATGGATTGGCTG	285	
CsGATA25 REV	TGGCTGATTGGTTATTGGGATCG		

CsGATA26 FOR	GAAATATCGGCTCTCGGAGTTTCG	240	
CsGATA26 REV	AATTGGAGGGAATTGATAAGGAAGC		
CpEF1a FOR	TTGACAACTGAAGTTAAGTCTGTTGAGATG	248	
CpEF1a REV	TGAGAGGTGTGGCAATCAAGCAC		
CpGATA1 FOR	ATGGGTATGATGGATGTCACACAAA	273	
CpGATA1 REV	GCCAGTGGAAGAAGAAGAAGTGG		
CpGATA2 FOR	ATGAAAGCAATCAAGAAGTGGAAACTGG	226	
CpGATA2 REV	GGTGACGGTGGCATCGGGTT		
CpGATA3 FOR	ATGGATGACCTTCTGGATTTCTCTTC	148	
CpGATA3 REV	GGTGGAAACCGGCGTTGAACT		
CpGATA4 FOR	GTGTTTGGAGGCTAAGGCTTTGAA	218	
CpGATA4 REV	ACTCTTCGTAATCGTCATCTTCTTGAAC		
CpGATA5 FOR	CGGAGATGTTATGGATGATGTCG	171	
CpGATA5 REV	AGTAAGAGCACGGCTTGTACCTTTT		
CpGATA6 FOR	TGGTAAATCAGAATCAGAGGAATGCTAT	225	
CpGATA6 REV	GAGACACTGTCATGGCTATACGAACC		
CpGATA7 FOR	AACAACTCACTCCTTCTTCCTCTTTCTC	148	
CpGATA7 REV	GAGACACTCCACCTTCCGAACTATC		
CpGATA8 FOR	TTATTCAGAGTCGTCCATCGTTCCT	149	
CpGATA8 REV	TACAGAGAGGCGAATTATGATTCCC		
CpGATA9 FOR	GAGGATAAGAAGAACAAAAGGAGCAA	209	
CpGATA9 REV	TTTCAAGCAAAAACAGAGCCATAT		
CpGATA10 FOR	CGATAATCAGGCGTTGGGGA	253	
CpGATA10 REV	AGTCGTTTTTGCACTTCCATAACCA		
CpGATA11 FOR	AACACGAGTATTCGCCCTCTCATT	184	
CpGATA11 REV	TTTCTTCTCCTCCTCCTCCTTGAG		
CpGATA12 FOR	CTGATTCCTCCACTCTCACTTTACTCG	246	
CpGATA12 REV	TTTGAAATTCTCTGGGTTTTGCG		
CpGATA13 FOR	ACAACAAGAACAGTTTAACAGGGACG	204	
CpGATA13 REV	TTGACAGTCTCGGGAAATTCAGC		
CpGATA14 FOR	GAACACGACCACGACGACTCTGT	219	
CpGATA14 REV	CTTTGACGATTTCACCAAGGAAGAA		
CpGATA15 FOR	CAGATGTTCAACGGAAACTACACGA	230	SYBR
CpGATA15 REV	CTTGTGAAGTCATGGACGAGGC		
CpGATA16 FOR	GAACAATAATCATGGGTTGGCGT	251	
CpGATA16 REV	GGGTTGAGGAGTGTGTACTGGTCAT		
CpGATA17 FOR	AAGGGCAATAATAGGAATATGGGAGA	183	
CpGATA17 REV	GTCTCAACAGGTGTGAACAAGACCAC		
CpGATA18 FOR	CGTCCCCCAATTCAGCTTATTTCC	149	
CpGATA18 REV	TTCGGCATTTTGTTCAAAGGTCG		
CpGATA19 FOR	TCCGAGGATGTCTTCGCCAG	230	
CpGATA19 REV	CACCGCCATTGCTGTTTCTGT		
CpGATA20 FOR	CTCCTTACCTCACGCTGCCG	232	
CpGATA20 REV	CGAACTGCTGCTACTGCTACTGCT		
CpGATA21 FOR	GCAGCAGACTACCTTCTTCTCCATG	201	
CpGATA21 REV	CGAGATAAATCCCAGGCGAAATTAG		
CpGATA22 FOR	ACTCCACCCAAGAAGAATACTCTGC	129	
CpGATA22 REV	TGAGATTAGATTCAAAATTCGGAAGG		
CpGATA23 FOR	CTCCAATTTCCAAGATGTGATGTACG	256	
CpGATA23 REV	ACCGTTTCCAATCAAAGGGTTGTA		
CpGATA24 FOR	TCTCTCTCAGGCATTTCATCAAACA	239	
CpGATA24 REV	GCGACAGTGGTGACAGGTCCT		
CpGATA25 FOR	GGCTGATGAGCATGAAAATGAAGG	164	
CpGATA25 REV	CGAAAACATAAACTTGACCCTGATAAGAC		
CpGATA26 FOR	GGAGCATTTCACCATTGATGATCTA	180	
CpGATA26 REV	CGAGAGCCGATATTTCCATTGA		
CpMAKR4 FOR	GCATTCTTCCCTTTGTCTCAAGC	171	TaqMan
CpMAKR4 REV	CTCTTTTCCAATTTAGCACCCTTCTT		
CpMAKR4 PROBE	**FAM**-ACCAAATCAGGTTGCTCAGAGGAGTCCACT-**BHQ1**		
CpEF1a FOR	TTGACAACTGAAGTTAAGTCTGTTGAGATG	148	
CpEF1a REV	CTGGGTCATCCTTGGAGTTTGAG		
CpEF1a PROBE	**FAM**-AACCACGCTTGAGATCCTTGACAGCAACG-**BHQ1**		

Supplementary Table S5. List of GATA genes in *Cucumis sativus* and *Cucurbita pepo.*

Name in *Cucumis sativus*	ID in databases			Chromosome Nr. and position on the chromosome	Name in *Cucurbita pepo*	Cucurbit Genomics Database (ID)	% amino acid identity to *C. sativus* GATA protein	% amino acid similarity to *C. sativus* GATA protein
	Phytozome (locus)	Cucurbit Genomics Database (Chinese Long cucumber)	NCBI GenBank (reference sequence)					
**CsGATA1**	No locus	Csa1G569090	XM_011661430.1	1 (20743377-20744463)	**CpGATA1**	Cp4.1LG04g08520	54%	66%
**CsGATA2**	Cucsa.397360	Csa1G587970	XM_004135770.1	1 (22118831-22120914)	**CpGATA2**	Cp4.1LG04g10690	59%	65%
**CsGATA3**	Cucsa.194390	Csa2G162660	XM_004150295.1	2 (9331162-9332655)	**CpGATA3**	Cp4.1LG12g01010	79%	83%
**CsGATA4**	Cucsa.109180.1 (.2;.3)	Csa2G251490.1 (.2)	XM_004150092.1	2 (12381477-12382571)	**CpGATA4**	Cp4.1LG16g02750<br>Cp4.1LG05g12840	78%<br>79%	84%<br>84%
**CsGATA5**	Cucsa.160520.1 (.2;.3;.4;.5;.6;.7)	Csa2G370420.1 (.2)	XM_011651386.1	2 (18237486-18243802)	**CpGATA5**	Cp4.1LG07g01700<br>Cp4.1LG11g04800	87%<br>82%	89%<br>84%
**CsGATA6**	Cucsa.160530	Csa2G370430	XM_004152556.1	2 (18245818-18251439)	**CpGATA6**	Cp4.1LG11g04800<br>Cp4.1LG07g01700	89%<br>86%	92%<br>90%
**CsGATA7**	Cucsa.161160	Csa2G373450	XM_011651894.1	2 (18666857-18668584)	**CpGATA7**	Cp4.1LG11g05190	55%	62%
**CsGATA8**	Cucsa.321290.1 (.2)	Csa3G017200.1 (.2)	XM_004147187.1	3 (1733153-1739264)	**CpGATA8**	Cp4.1LG08g07130<br>Cp4.1LG14g09160	82%<br>76%	87%<br>79%
**CsGATA9**	Cucsa.252730	Csa3G165640	XM_004134187.1	3 (10938002-10938626)	**CpGATA9**	Cp4.1LG08g05190<br>Cp4.1LG03g11690	90%<br>87%	93%<br>89%
**CsGATA10**	Cucsa.038810	Csa3G457670	XM_004150555.1	3 (20795117-20796204)	**CpGATA10**	Cp4.1LG02g14590<br>Cp4.1LG03g14100	78%<br>75%	86%<br>81%
**CsGATA11**	Cucsa.340570	Csa3G843820	XM_004141093.1	3 (34100298-34101252)	**CpGATA11**	Cp4.1LG08g06520	73%	78%
**CsGATA12**	Cucsa.312530	Csa3G895650	XM_011654312.1	3 (38551617-38552757)	**CpGATA12**	Cp4.1LG06g03840	65%	69%
**CsGATA13**	Cucsa.313750	Csa3G912920	XM_004148334.1	3 (39577751-39583101)	**CpGATA13**	Cp4.1LG15g03070<br>Cp4.1LG05g02270	83%<br>88%	87%<br>91%
**CsGATA14**	Cucsa.201200	Csa4G043890	XM_004146437.1	4 (3394791-3395838)	**CpGATA14**	Cp4.1LG14g01630	70%	77%
**CsGATA15**	Cucsa.106490	Csa4G046650	XM_004146505.1	4 (3624324-3625837)	**CpGATA15**	Cp4.1LG01g07010	69%	73%

**CsGATA16**	Cucsa.153540	Csa4G286370	XM_011656060.1	4 (11065621-11066229)	**CpGATA16**	Cp4.1LG16g09100<br>Cp4.1LG00g11890	65%<br>65%	68%<br>68%
**CsGATA17**	Cucsa.353330	Csa4G646060	XM_011655871.1	4 (21924862-21926381)	**CpGATA17**	Cp4.1LG02g06040	truncated	truncated
**CsGATA18**	Cucsa.308400	Csa5G622830.1 (.2)	XM_004142378.1	5 (24736218-24738104)	**CpGATA18**	Cp4.1LG09g04140<br>Cp4.1LG01g14740	81%<br>85%	86%<br>88%
**CsGATA19**	Cucsa.069460	Csa6G312540	XM_004150033.1	6 (14872405-14873032)	**CpGATA19**	Cp4.1LG12g03020	87%	90%
**CsGATA20**	Cucsa.137630	Csa6G405920.1 (.2)	XM_004149856.1	6 (18352694-18354402)	**CpGATA20**	Cp4.1LG01g24410	65%	72%
**CsGATA21**	Cucsa.127470	Csa6G502700	XM_004143467.1	6 (25337463-25338194)	**CpGATA21**	Cp4.1LG19g09270	80%	86%
**CsGATA22**	Cucsa.043350	Csa6G504690	XM_011659733.1	6 (25624904-25625684)	**CpGATA22**	Cp4.1LG10g00520	60%	65%
**CsGATA23**	Cucsa.218190	Csa7G064580	XM_011660512.1	7 (3848829-3853491)	**CpGATA23**	Cp4.1LG04g07180<br>Cp4.1LG15g03820	81%<br>68%	84%<br>71%
**CsGATA24**	Cucsa.205230	Csa7G405980	XM_011661080.1	7 (15586933-15587971)	**CpGATA24**	Cp4.1LG17g07330	56%	61%
**CsGATA25**	Cucsa.359570.1 (.5;.6)	Csa7G447800.1	XM_011660240.1	7 (18027551-18032452)	**CpGATA25**	Cp4.1LG12g09700<br>Cp4.1LG17g04450	90%<br>91%	92%<br>93%
**CsGATA26**	Cucsa.361150	Csa7G452960	XM_004141609.1	7 (919135736-19136886)	**CpGATA26**	Cp4.1LG17g05330<br>Cp4.1LG12g08350	85%<br>85%	91%<br>90%

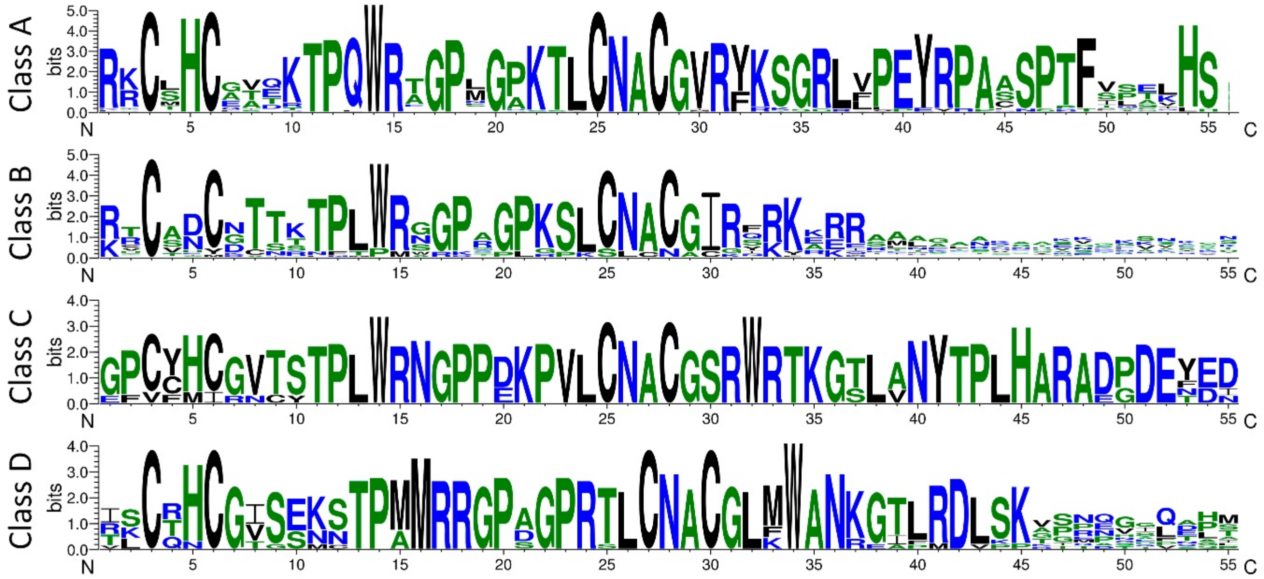

Supplementary Figure S1. Sequence logos of different classes of GATA domains from *Arabidopsis thaliana*, *Cucumis sativus* and *Cucurbita pepo*.

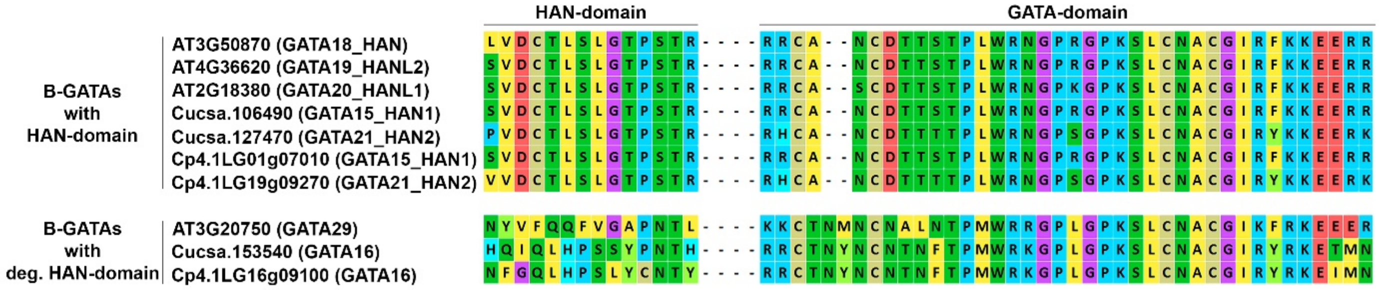

Supplementary Figure S2. HAN-domain and GATA-domain sequencealignment of the B class GATA proteins. Amino acid sequences from *Arabidopsis thaliana* (AT), *Cucumis sativus* (Cucsa) and *Cucurbita pepo* (Cp) were used.

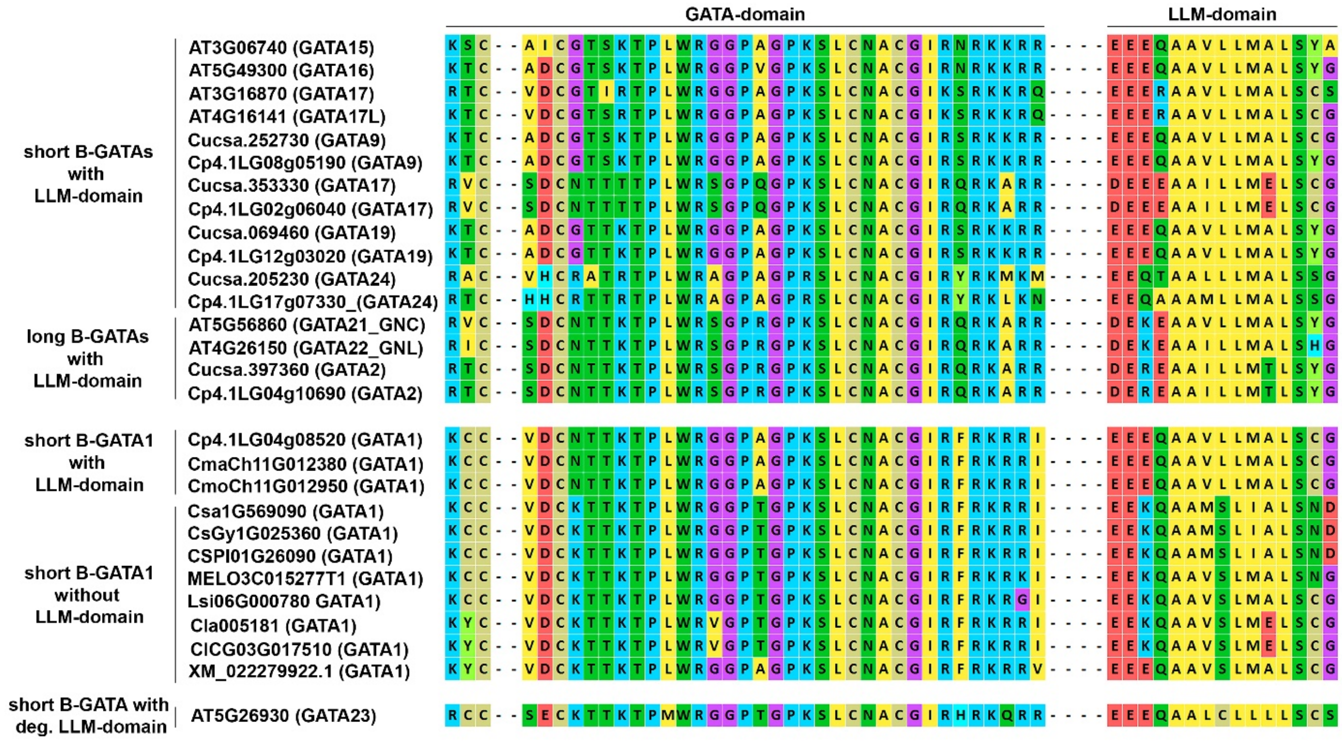

Supplementary Figure S3. GATA-domain and LLM-domain sequences alignment of the B class GATA proteins. Amino acid sequences from *Arabidopsis thaliana* (AT), *Cucumis sativus* (Cucsa/Csa/Cs/CS), *Cucurbita pepo* (Cp), *Cucurbita maxima* (Cma), *Cucurbita moschata* (Cmo), *Cucumis melo* (MELO), *Lagenaria siceraria* (Lsi), *Citrullus lanatus* (Cla/Cl) and *Momordica charantia* (XM) are represented.

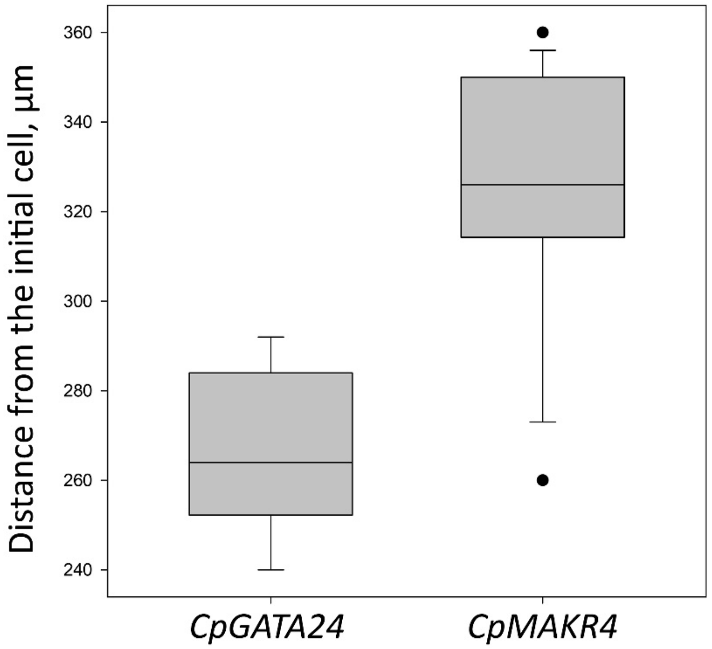

Supplementary Figure S4. The distance from the initial cells in *Cucurbita pepo* root meristem to the first cell in a protoxylem file expressing *CpGATA24* or *CpMAKR4*.
Whiskers (error bars) above and below the box indicate the 90th and 10th percentiles. Dots represent outliers. Statistical analysis was performed using the Student's T-test after a Shapiro-Wilk normality test (P = 0.129, statistically significant difference between the groups (P <0.001). Numbers of analyzed transgenic roots: *CpGATA24* – n=10, *CpMAKR4* – n=14.

References for Supplementary Material

Hornung, E., Krueger, C., Pernstich, C., Gipmans, M., Porzel, A., and Feussner, I. (2005). Production of (10*E*,12*Z*)-conjugated linoleic acid in yeast and tobacco seeds. *Biochimica et Biophysica Acta (BBA) - Molecular and Cell Biology of Lipids* 1738(1–3), 105-114. doi: 10.1016/j.bbalip.2005.11.004

Shaner, N. C., Lambert, G. G., Chammas, A., Ni, Y., Cranfill, P. J., Baird, M. A., et al. (2013). A bright monomeric green fluorescent protein derived from *Branchiostoma lanceolatum*. *Nature Methods* 10(5), 407-409. doi: 10.1038/nmeth.2413
